# Modulation of defect-mediated energy transfer from ZnO nanoparticles for the photocatalytic degradation of bilirubin

**DOI:** 10.3762/bjnano.4.81

**Published:** 2013-11-04

**Authors:** Tanujjal Bora, Karthik Kunjali Lakshman, Soumik Sarkar, Abhinandan Makhal, Samim Sardar, Samir Kumar Pal, Joydeep Dutta

**Affiliations:** 1 Center of Excellence in Nanotechnology, School of Engineering and Technology, Asian Institute of Technology, P. O. Box 4, Klong Luang, Pathumthani – 12120, Thailand; 2 Chair in Nanotechnology, Water Research Center, Sultan Qaboos University, P. O. Box 17, Al Khoud – 123, Sultanate of Oman; 3 Department of Chemical, Biological & Macromolecular Sciences, Unit for Nanoscience & Technology, S. N. Bose National Centre for Basic Sciences, J D Block, Sector III, Salt Lake, Kolkata – 700 098, India

**Keywords:** bilirubin, Förster resonance energy transfer (FRET), neonatal jaundice, oxygen vacancy, photocatalysis, phototherapy, zinc oxide nanoparticles

## Abstract

In recent years, nanotechnology has gained significant interest for applications in the medical field. In this regard, a utilization of the ZnO nanoparticles for the efficient degradation of bilirubin (BR) through photocatalysis was explored. BR is a water insoluble byproduct of the heme catabolism that can cause jaundice when its excretion is impaired. The photocatalytic degradation of BR activated by ZnO nanoparticles through a non-radiative energy transfer pathway can be influenced by the surface defect-states (mainly the oxygen vacancies) of the catalyst nanoparticles. These were modulated by applying a simple annealing in an oxygen-rich atmosphere. The mechanism of the energy transfer process between the ZnO nanoparticles and the BR molecules adsorbed at the surface was studied by using steady-state and picosecond-resolved fluorescence spectroscopy. A correlation of photocatalytic degradation and time-correlated single photon counting studies revealed that the defect-engineered ZnO nanoparticles that were obtained through post-annealing treatments led to an efficient decomposition of BR molecules that was enabled by Förster resonance energy transfer.

## Introduction

Bilirubin (BR) is a yellow-orange pigment which is a byproduct of the normal heme catabolism in mammals. In the human body, 250–400 mg BR is produced every day [1] and can exist in the body both as a free molecule and as an albumin complex. The unconjugated (*Z*,*Z*)-BR isomer is insoluble in water and is converted in the liver into the water-soluble (*Z*,*E*)-BR isomer with the assistance of glucuronic acid. Most of the BR is then extracted in the bile while a small portion is excreted in the urine [2]. Excess bilirubin in blood can lead to deposits on tissues, which gives rise to neurotoxicity and hyperbilirubinemia and/or a yellowish pigmentation of the skin, a disease commonly known as jaundice. According to the World Health Organization, almost 30,000 people suffering from jaundice die every year [3]. Jaundice is most commonly seen in newly born babies (neonatal jaundice) and typically develops within a few weeks after birth. There can be many sources for neonatal jaundice, such as weak liver function, a high level of red blood cells, and the deficiency of important enzymes in the body [4]. At present, phototherapy, the treatment of various diseases with light irradiation, is the most widely used treatment for neonatal jaundice. In it, the unconjugated (*Z*,*Z*)-BR isomers are converted to water-soluble (*Z*,*E*)-BR isomers by using a light source. Additionally, upon absorption of light, the (*Z*,*Z*)-BR isomers can also react with oxygen in the blood to form colorless oxidation products, which are typically excreted in the urine [2]. However, the photo-oxidation of (*Z*,*Z*)-BR is a slow process and the isomerization of (*Z*,*Z*)-BR occurs much faster than the photo-oxidation [5].

In recent years, the use of nanotechnology in medical science is gaining a lot of attention across the world. Research that focuses on the use of various nanostructured materials in different areas, such as for drug delivery [6], cancer treatments [7–8], etc. is underway. Out of the numerous nanostructured materials, zinc oxide (ZnO) is one of the most promising materials for applications in the medical field, because of its biocompatibility, biodegradability and non-toxicity [9]. Moreover, ZnO can degrade various organic compounds efficiently through photocatalysis [10–11]. It has been reported that the native defects in the ZnO lattice, mostly the oxygen vacancy sites, play an important role in the photocatalytic activity of the nanostructures [11]. Oxygen vacancies have been reported as the cause of the characteristic green luminescence of ZnO [12–14]. These vacancies exist in three charged states: singly charged (V_O_^+^), doubly charged (V_O_^++^) and neutral (V_O_^x^). The presence of the oxygen vacancies and other native defects in the ZnO lattice reduces the direct e^−^/h^+^ recombination process and thus increases the quantum yield of ZnO nanocrystalline photocatalysts. Furthermore, the defect-mediated emission of energy from the ZnO nanostructures can also effectively degrade various organic compounds in water through Förster resonance energy transfer (FRET) [15]. Hence, for an efficient photocatalysis system that is based on ZnO nanostructures, the control of such defect sites is a crucial factor. One effective way to modulate the concentration of defects in the ZnO lattice is to anneal the ZnO nanostructures in an oxygen-rich atmosphere. The effect of annealing on the native defects of ZnO has been studied extensively and it has been demonstrated that the crystallinity of ZnO can be improved by annealing the samples at higher temperatures, typically above 500 °C [16–20]. However, some studies show that annealing at higher temperatures can also introduce defects, such as oxygen interstitials in the ZnO lattice [21–22].

In the present work, we have explored the potential use of ZnO nanoparticles as a phototherapy agent to efficiently degrade BR molecules by controlling the surface defect-states of the nanoparticles through annealing in an oxygen-rich atmosphere. In one of our recent studies, it was demonstrated that upon surface adsorption of BR molecules on ZnO nanostructures, a resonant defect-mediated energy transfer from the photo-excited ZnO nanostructures to the BR molecules induces their photodegradation [15]. It was also demonstrated that the system can effectively degrade BR when it is bound to albumin. Although literature related to the molecular transformation of water-insoluble BR through photocatalysis is limited, a few studies are available on the photocatalytic degradation of BR adsorbed on nanostructured hydroxyapatite coatings [23] or molecularly imprinted titania films [24]. The current study focuses on the efficient utilization of the defect-mediated emission from the ZnO nanoparticles. The emission is modulated through annealing of the nanoparticles, in order to improve the overall efficiency of the FRET process and hence to expedite the photocatalytic degradation of BR molecules. By using steady state and picosecond-resolved fluorescence spectroscopy, we have explored the influence of the surface defect-states (mainly the oxygen vacancies) of the various as-synthesized and annealed ZnO nanoparticles on the photocatalytic degradation process of BR.

## Results and Discussion

**Figure 1:**
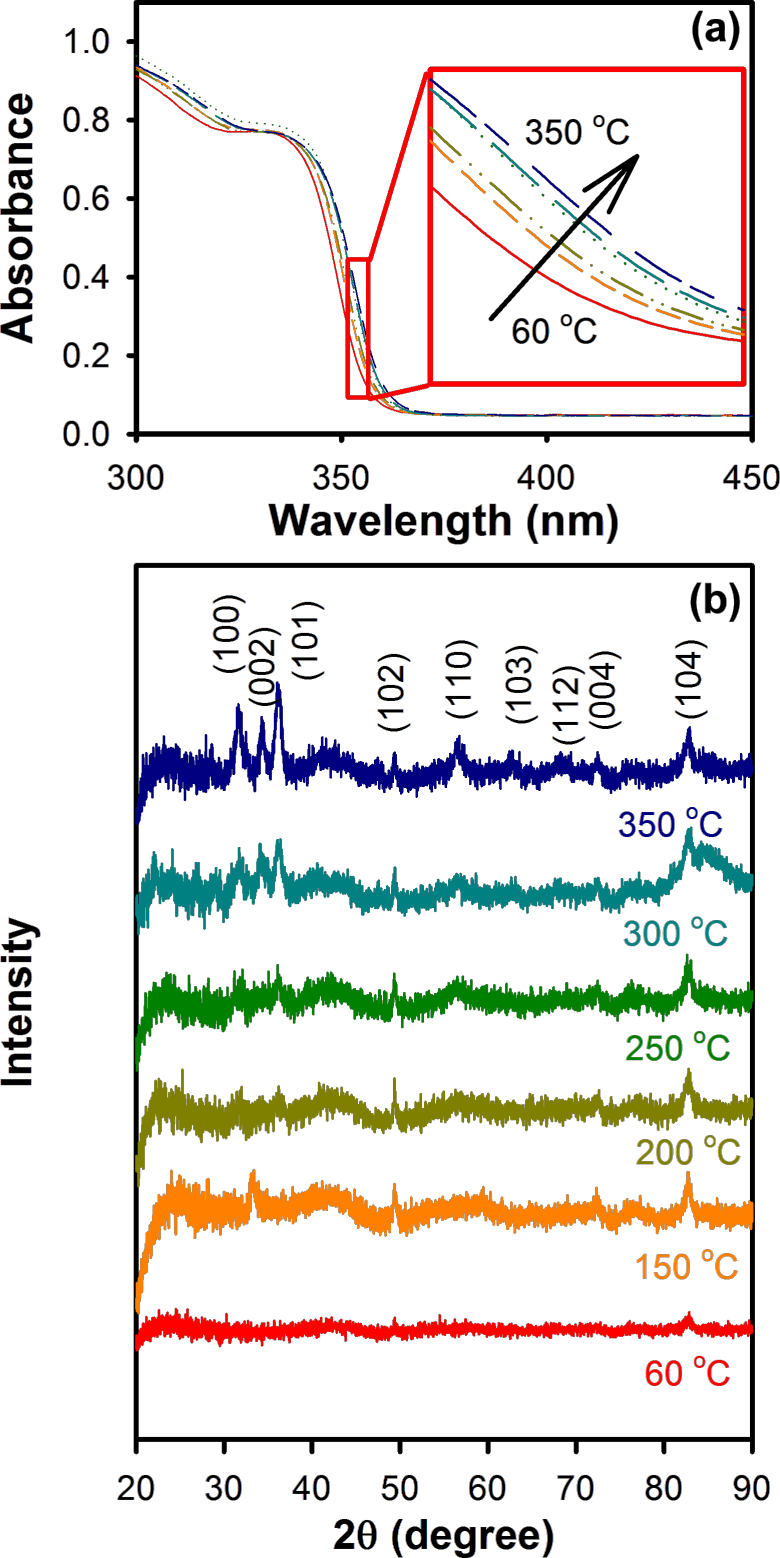
(a) UV–vis optical absorption and (b) X-ray diffraction pattern of ZnO nanoparticles annealed at various temperatures in air for 1 h. The 60 °C sample indicates the non-annealed as-synthesized ZnO nanoparticles.

Figure 1a shows the UV–vis optical absorption spectra of the as-synthesized ZnO nanoparticles and the particles annealed in air at different temperatures. The as-synthesized ZnO nanoparticles show a sharp absorbance onset at about 340 nm, which indicates an almost uniform size of the nanoparticles. However, upon annealing at higher temperatures, a slight red-shift in the absorption spectra of the nanoparticles was observed, which indicates a marginal increase in the size of the ZnO nanoparticles after annealing [25]. The changes in the crystallite size of the nanoparticles after annealing were estimated from their respective XRD patterns (Figure 1b) by using the Scherrer equation and the results are shown in Table S1 (Supporting Information File 1). The crystallite size of the as-synthesized ZnO nanoparticles was found to be about 7.3 nm and marginally increases to 7.8 nm upon annealing at 250 °C.

**Figure 2:**
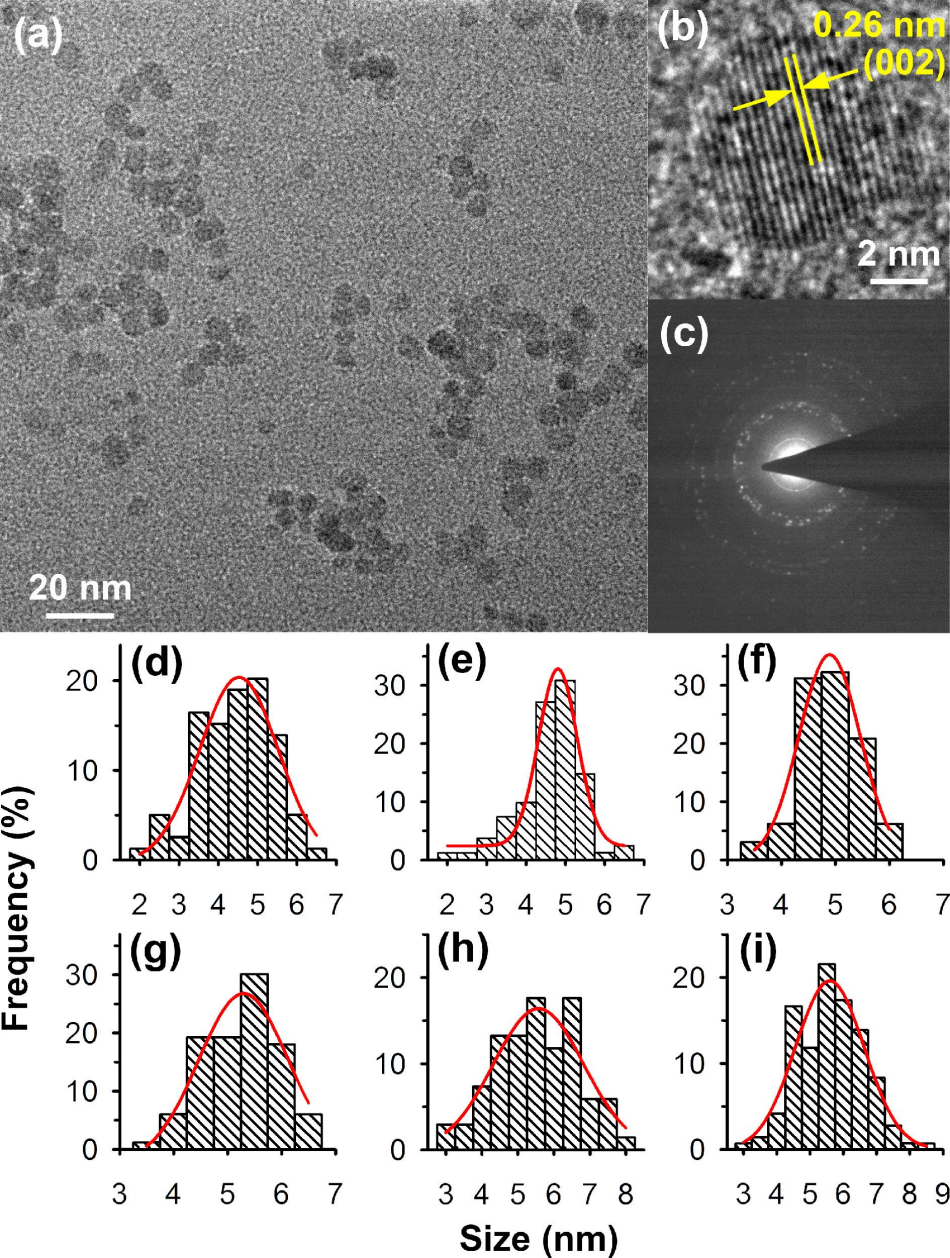
(a) Transmission electron micrograph, (b) high resolution TEM image of a single ZnO nanoparticle and (c) SAED pattern of the ZnO nanoparticles annealed at 250 °C. The particle size distribution of the (d) as-synthesized, (e) 150 °C, (f) 200 °C, (g) 250 °C, (h) 300 °C and (i) 350 °C annealed ZnO nanoparticles are also shown.

The changes in the sizes of ZnO nanoparticles were further verified by transmission electron microscopy (TEM) analysis. Figure 2a shows the typical TEM image of the fairly monodispersed ZnO nanoparticles annealed at 250 °C. As shown in Figure 2b, the lattice spacing of 0.26 nm indicates the (002) plane of the wurtzite structure of ZnO nanoparticles. The polycrystalline nature of the nanoparticles is confirmed by the corresponding selected area electron diffraction (SAED) pattern (Figure 2c). The particle size distributions of all samples obtained from the respective TEM micrographs are also shown in Figure 2d–i. For the as-synthesized ZnO nanoparticles the mean particle size was found to be 4.5 nm, while the nanoparticles increased in size upon annealing at higher temperatures, which corroborates well with our previous observations from XRD analysis. The mean size for the ZnO nanoparticles annealed at 250 °C was obtained to be 5.5 nm. The maximum mean particle size of 5.9 nm was obtained in the case of the nanoparticles annealed at 350 °C.

Here, it should be noted that the size of the nanoparticles estimated by using the Scherrer equation yielded larger crystallite sizes than the size obtained from TEM images. This discrepancy arises because of a line broadening in XRD patterns that originate primarily from the inhomogeneous micro-strain of the samples as well as effects from the measurement apparatus [26]. As a result, the size estimation from XRD peaks using the Scherrer equation typically yields larger sizes than the actual sample size that can be obtained from TEM more accurately.

**Figure 3:**
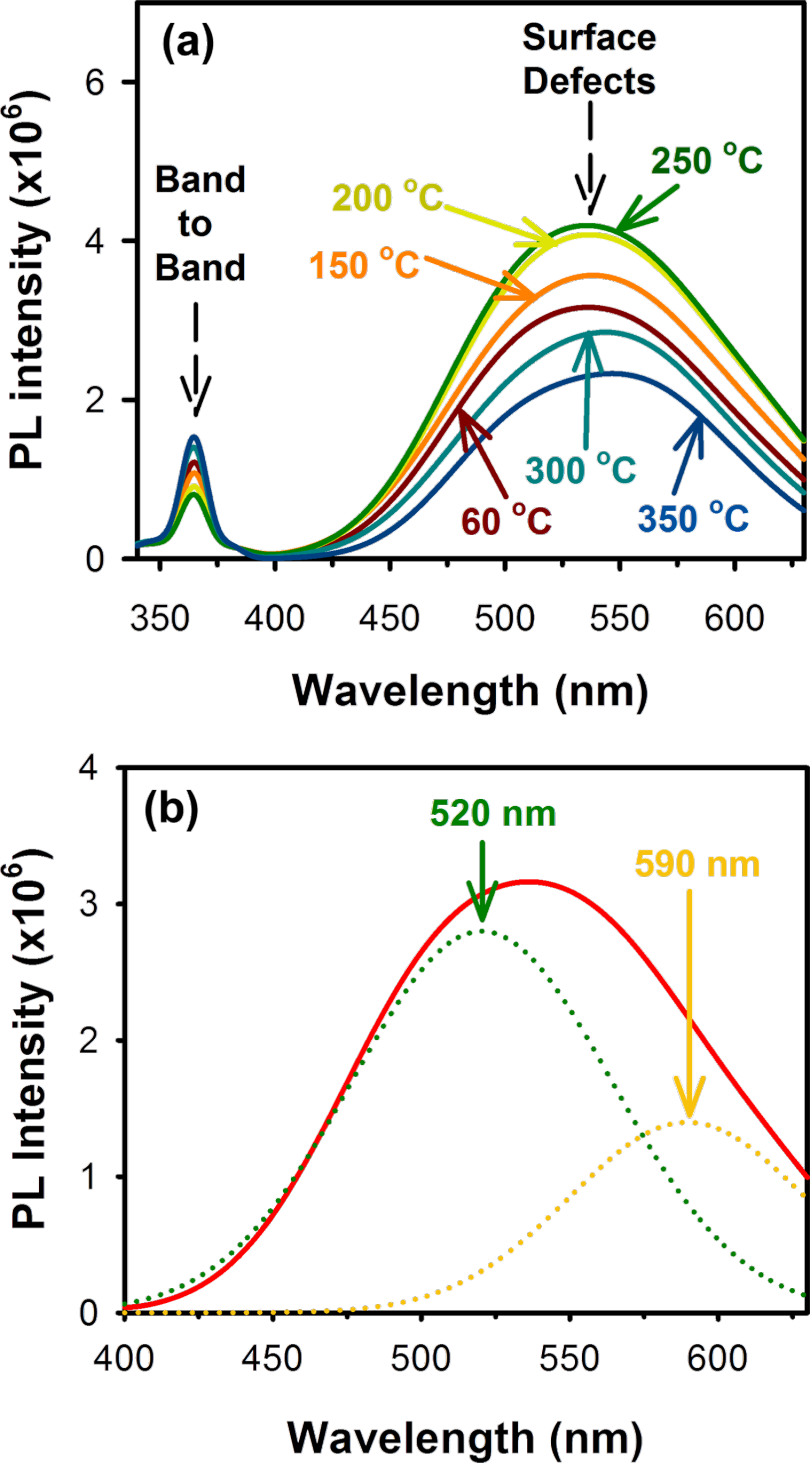
(a) Room temperature photoluminescence (PL) spectra of the ZnO nanoparticles annealed at various temperatures in air for 1 h (excitation wavelength: 320 nm) and (b) defect mediated green–yellow emission from ZnO nanoparticles showing the two individual emission bands peaking at 520 nm and 590 nm, which are contributed by the singly charged and doubly charged oxygen vacancy states respectively.

The room temperature photoluminescence (PL) spectra of annealed ZnO nanoparticles are shown in Figure 3a. All the nanoparticle samples show a small UV emission at approx. 355 nm, which can be attributed to the near band edge transitions in the ZnO nanoparticles, and a large and broad green–yellow emission centered at around 530 nm, which can be attributed to the oxygen vacancy defect states (mostly present at the surface of the nanoparticles) [27]. A. van Dijken et al. [28] have proposed the transition of a photo-excited electron from the conduction band of ZnO to a deep-level trap state (V_O_^++^) as the origin of the green luminescence. Whereas Vanheusden et al. [29] reported that the recombination of an electron from a V_O_^+^ state to a valence band hole lead to the green luminescence peak. In contrast, J. D. Ye et al. [27] has reported that both the above assumptions are correct and demonstrated that the broad green–yellow luminescence from ZnO is mainly composed of two individual emission bands centered at approx. 520 and approx. 590 nm respectively, as shown in Figure 3b.

It was observed that with increasing annealing temperature the green–yellow emission from the ZnO nanoparticles also increases, which shows a rise in the number of surface defect-states. The maximum defect-originated emission was observed from ZnO nanoparticles annealed at 250 °C. These observations show that upon annealing the ZnO nanoparticles up to 250 °C in air, the concentration of the surface-defect sites in the nanoparticles gradually increases. Previously it was observed that the ZnO nanoparticles grow slowly along with the annealing temperatures, which demonstrates a gradual improvement of their crystallinity, as can be evidenced from TEM and XRD analysis respectively. However, at the same time, they also show higher defect densities near their surface for annealing temperatures up to 250 °C. In case of the as-synthesized ZnO nanoparticles, initially defects are randomly created within the particles as they grow and hence defects are located randomly from the core to the surface of the particles. As the particles are subjected to high-temperature annealing, the defects within the particles start to diffuse inside out, i.e., from the core of the particles towards the surface, increasing the defect densities near the surface of the particles. This results in a gradual increase of the surface-defect mediated green–yellow emission peak. It should be noted that during the process the core of the particles gets annealed first; however, the total defect concentration within the particle volume remains almost similar since no sintering of the particles was observed upon annealing at such low temperatures. Although the ZnO nanoparticles annealed at 250 °C demonstrated a slightly higher crystallite size (5.5 nm) than the as-synthesized nanoparticles (4.5 nm), only a marginal improvement in the crystallinity of the nanoparticles was observed from the XRD pattern compared to the as-synthesized particles because of the relatively constant total defect concentration within the particle volume.

For the ZnO nanoparticles annealed at temperatures above 250 °C, a significant drop in the surface-defect mediated emission band was observed, which indicates a reduction in the oxygen vacancy states. In this regard, Wei et al. [30] have recently reported that at annealing temperatures above 300 °C, oxygen vacancies can be eliminated from the ZnO crystal lattice and the Zn–O bonding can be enhanced, which confirms the observed reduction in the intensity of the green–yellow emission band from samples annealed at temperatures above 250 °C. Improvement in the ZnO crystal stoichiometry upon annealing at temperatures above 250 °C are also evident from the higher band to band emission of the annealed nanoparticles (Figure 3a) as well as from their respective XRD spectra (Figure 1b).

**Figure 4:**
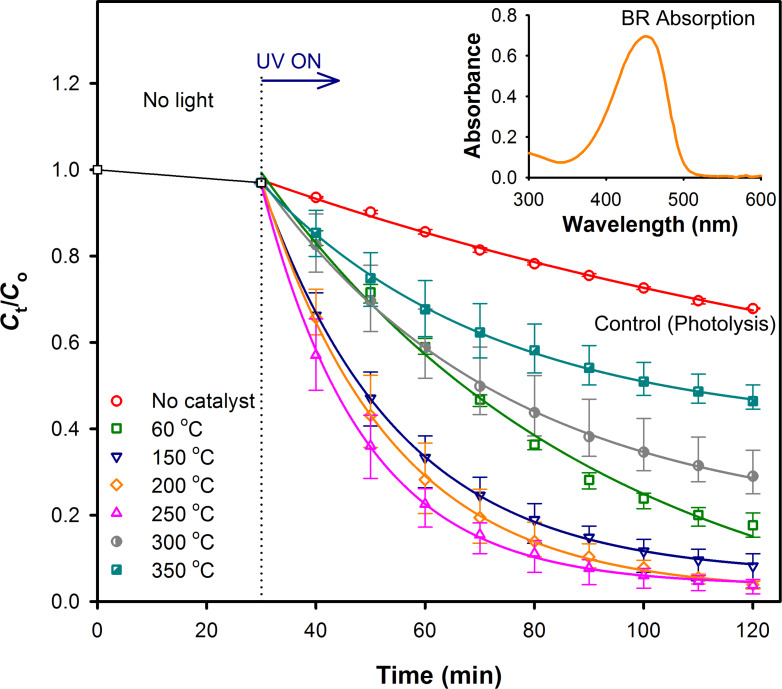
Relative concentration (*C*_t_/*C*_0_) of BR as a function of the UV irradiation time during photocatalytic degradation (monitored for BR peak absorbance at 450 nm) in the absence (control) and presence of ZnO nanoparticle catalysts air annealed at different temperatures. The adsorption of BR on the ZnO surface under no light condition was carried out for 30 min prior to the UV light irradiation. The inset shows the UV–vis absorption spectrum of the BR solution.

Following the optical characterization of the ZnO nanoparticle samples, we have explored the effect of the concentration of the surface defects in the ZnO nanoparticles on the photocatalytic degradation of BR, when using the nanoparticles as a photocatalyst medium. The photocatalytic degradation of BR was conducted by using a 7 μM BR solution prepared in chloroform as the test sample, which shows an absorption peak at 450 nm (inset of Figure 4, see below). Under UV light irradiation, the photocatalytic degradation of BR in the presence and in the absence of different ZnO nanoparticle samples was studied by constantly monitoring the absorption spectra of BR solution at 450 nm wavelength over a period of 90 min. The BR adsorption on the ZnO surface in dark was studied over a period of 1 h and it was found that irrespective of the annealing temperatures an equilibrium adsorption is reached after about 30 min. All the adsorption curves were found to be of the Langmuir type. The detailed analysis of BR adsorption in dark is described in the Supporting Information File 1 (Figure S2).

Figure 4 shows the relative concentration (*C*_t_/*C*_0_) of BR with respect to the UV irradiation time after a BR adsorption period in dark of 30 min. A control experiment was performed in the absence of ZnO nanoparticles and the photolytic degradation of BR was monitored. In the absence of a catalyst, a degradation of the BR concentration of about 30% was observed upon UV light irradiation for 90 min. However, in the presence of the ZnO nanoparticles as the catalyst medium, a much faster degradation of BR was observed. In this context, it has been previously shown that the defect-mediated energy transfer from the surface defects of ZnO nanoparticles to the BR molecules through the FRET mechanism is the pre-dominant reason for the faster degradation of BR molecules under UV irradiation in the presence of ZnO nanoparticles [15].

Upon inclusion of the as-synthesized ZnO nanoparticles (hydrolyzed at ≈60 °C) in BR, almost 50% degradation was observed to occur within 40 min of UV irradiation, leading to an about 70% faster photocatalytic reduction of BR compared to the degradation of BR in 40 minutes when no catalyst was used. The reduction in the BR concentration increased further when annealed ZnO nanoparticles (up to 250 °C) were used as the photocatalysts because of their higher concentrations of surface defects. The rates of the photocatalytic degradation of BR were found to follow a first-order exponential equation with a maximum photocatalytic activity for the ZnO nanoparticles annealed at 250 °C. However, when the surface defects were reduced by annealing the ZnO nanoparticles at temperatures above 250 °C, a significant drop in the catalytic activity was observed, which suggests the vital role of the surface defects in the photocatalytic degradation of BR. It should be noted that the FRET process between the ZnO nanoparticles and the BR molecules does not interfere with the normal phototherapy process of BR under UV irradiation. Hence, the photoproducts formed after the photocatalysis of BR in the presence of ZnO nanoparticles should mainly contain the structural (*Z*-lumirubin) and configurational ((*Z*,*E*)-BR) isomers of water-insoluble BR, which are the usual photoproducts of the BR phototherapy [2]. In addition, the presence of methylvinylmaleimide (MVM) as an outcome of the photocatalytic degradation of BR in the presence of ZnO nanoparticles has also been evidenced in our previous study [15].

In order to obtain further insights on the influence of the surface defect concentration of the ZnO nanoparticle samples on the BR photocatalytic degradation rate (*R*_0_), we conducted photocatalysis experiments with different initial concentrations (*C*_0_) of BR, while keeping the ZnO concentration constant. For these experiments, we selected the as-synthesized ZnO nanoparticles, the ZnO nanoparticles annealed at 250 °C, which had the maximum number of surface defect-states and the ZnO nanoparticles annealed at 350 °C, which have a minimum number surface defect-states. The rate of the photocatalytic degradation, *R*_0_, was then fitted by using the Langmuir–Hinshelwood (L–H) kinetic model, which typically explains the bimolecular reaction of two species upon surface adsorption [31].

**Figure 5:**
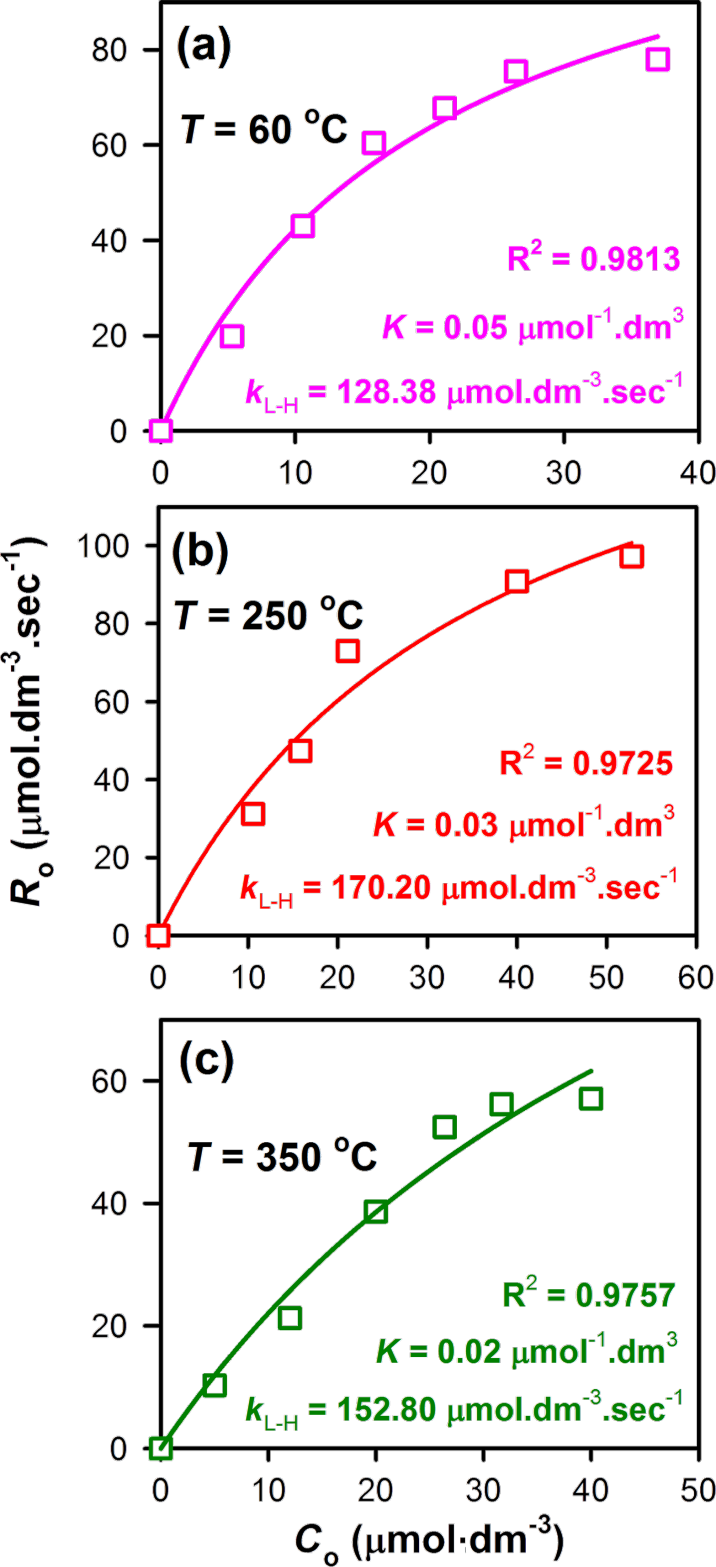
Langmuir–Hinshelwood (L–H) plot showing the Langmuir–Hinshelwood rate constant (*k*_L–H_), Langmuir adsorption coefficient (*K*) and regression coefficients (R*^2^*) for the photocatalytic reactions conducted by using (a) the as-synthesized, (b) 250 °C and (c) 350 °C annealed ZnO nanoparticles as catalysts.

Upon fitting the rate constant (*R*_0_) vs the initial concentration of BR (*C*_0_) curves using Equation 1 (see Experimental section), we observe that the photocatalytic degradation of BR exactly follows the phenomenon explained by the L–H model (Figure 5). In case of the as-synthesized ZnO nanoparticles, the value of the L–H rate constant (*k*_L–H_) was found to be 128.38 μmol·dm^−3^·s^−1^; which then improved to170.20 μmol·dm^−3^·s^−1^ for the 250 °C annealed ZnO nanoparticles. For the ZnO nanoparticles with the minimum surface defect-states, the values of *k*_L–H_ and *K* were found to decrease again to 152.80 μmol·dm^−3^·s^−1^ and 0.02 μmol^−1^·dm^3^, respectively.

**Figure 6:**
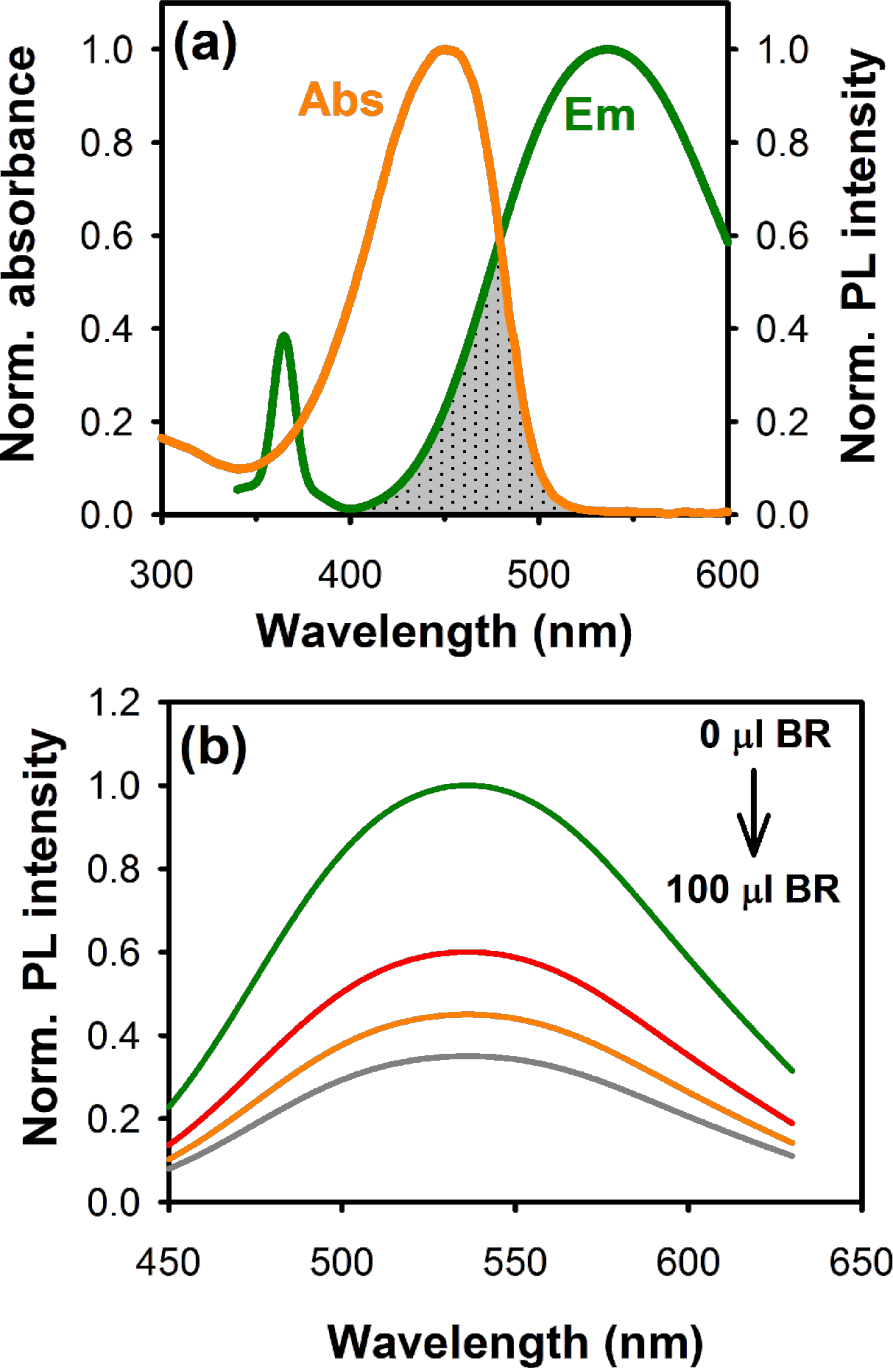
(a) Spectral overlap (shaded area) between the defect-mediated ZnO nanoparticle emission and the BR absorption and (b) quenching of the steady-state defect-mediated PL of the ZnO nanoparticles after gradual addition of BR into the system.

The energy transfer process between the acceptor BR molecules and the donor ZnO nanoparticles was then probed by studying the FRET dynamics process through picosecond-resolved fluorescence spectroscopy. For an efficient FRET process it is important to have a significant spectral overlap between the emission spectrum of the donor and the absorption spectrum of the acceptor species. There are generally two mechanisms used to describe the transfer of excitations between a donor and an acceptor species, namely Dexter and Förster mechanisms. In both of these mechanisms, the energy transfer rate is dependent on the distance between the donor and acceptor as well as the spectral overlap of the emission from donor and the absorption of acceptor molecules. However, the basic difference between the two mechanisms is that in the Dexter mechanism the donor and the acceptor exchange their electron, whereas in the Förster mechanism energy (not electrons) is exchanged between the donor and acceptor. Therefore, the overlap of wavefunctions or molecular orbitals between the donor and acceptor is very crucial in the case of the Dexter mechanism so that the electrons can occupy the molecular orbital of the other molecule. The energy transfer rate also decays exponentially with the distance between the donor and acceptor molecules, and the exchange normally occurs when the distance is smaller than 1 nm. On the other hand, the energy transfer of the Förster mechanism is highly influenced by the Förster distance (*R*_0_) and the relative orientation of the transition dipoles of the donor and acceptor in space. Furthermore, the energy exchange occurs within a minimal donor–acceptor distance of 1–10 nm. Therefore here in this study, the consideration of Dexter mechanism (orbital overlap) via an exchange process associated with the transfer of single charge carriers can be excluded, as it is a short-range mechanism. Figure 6a shows the spectral overlap of the defect-mediated ZnO emission and the BR absorption, which is responsible for the energy exchange process between donor ZnO and acceptor BR. As shown in Figure 6b, the defect mediated green–yellow emission of the ZnO nanoparticles was found to be suppressed upon gradual addition of BR into the system. This clearly indicates that an energy exchange process takes place through FRET when the two species are in close proximity (typically 1–10 nm).

**Figure 7:**
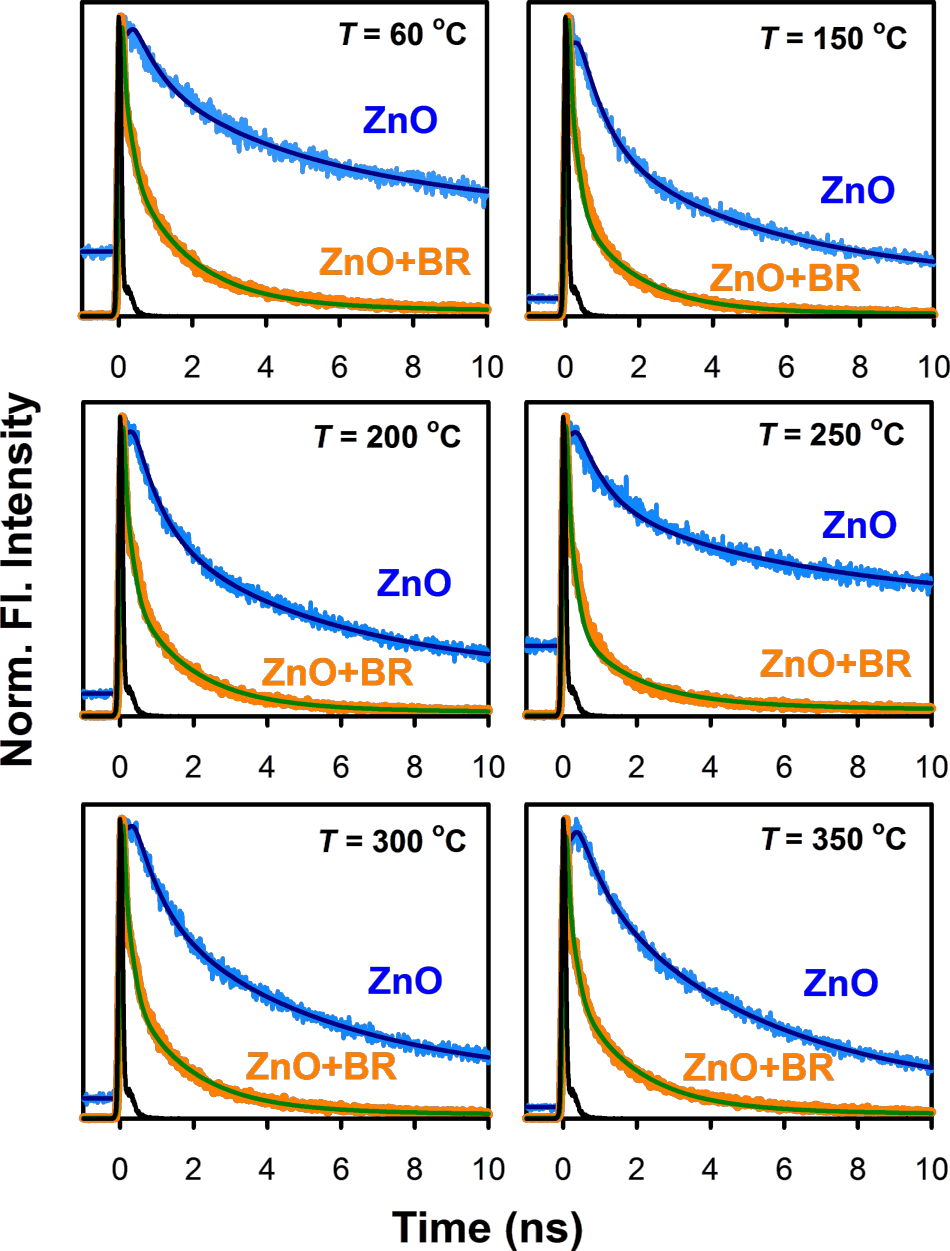
The picosecond-resolved fluorescence transients of the as-synthesized and annealed ZnO nanoparticles in the presence and in the absence of BR. The fluorescence decay was monitored at 540 nm with an excitation wavelength of 375 nm.

The fluorescence decay kinetics of the as-synthesized and annealed ZnO nanoparticles in the presence and in the absence of BR were then studied by using the picosecond-resolved TCSPC technique. The emissions from ZnO nanoparticles were detected at 540 nm with a laser excitation wavelength of 375 nm. As shown in Figure 7, upon addition of BR into the system, a faster fluorescence decay component was observed, attributed to the energy transfer process between ZnO and BR via FRET. The various decay transients of the ZnO nanoparticles in the presence and absence of BR, obtained after the deconvolution of the fluorescence decay curves with the instrumental response function (IRF, fitted globally by keeping the time scale fixed) are tabulated in Table 1.

**Table 1:** Picosecond-resolved fluorescence transients of the as-synthesized and annealed ZnO nanoparticles in the presence and in the absence of BR^a^.

annealing temperature (°C)	sample	τ_1_ (ns)	τ_2_ (ns)	τ_3_ (ns)	τ_avg_ (ns)	*E* (%)

60	ZnO only	0.71 (33%)	4.69 (46%)	39.20 (21%)	8.78	—
60	ZnO + BR	0.13 (72%)	1.60 (26%)	14.20 (2%)	0.79	91.53
150	ZnO only	0.71 (50%)	4.69 (43%)	39.20 (7%)	5.11	—
150	ZnO + BR	0.13 (81%)	1.60 (18%)	14.20 (1%)	0.53	88.63
200	ZnO only	0.71 (47%)	4.69 (45%)	39.20 (8%)	5.58	—
200	ZnO + BR	0.13 (78%)	1.60 (21%)	14.20 (1%)	0.58	86.20
250	ZnO only	0.71 (42%)	4.69 (33%)	39.20 (25%)	11.64	—
250	ZnO + BR	0.13 (85%)	1.60 (14%)	14.20 (1%)	0.48	93.79
300	ZnO only	0.71 (41%)	4.69 (52%)	39.20 (7%)	5.47	—
300	ZnO + BR	0.13 (78%)	1.60 (21%)	14.20 (1%)	1.85	88.18
350	ZnO only	0.71 (28%)	4.69 (67%)	39.20 (5%)	5.30	—
350	ZnO + BR	0.13 (75%)	1.60 (23%)	14.20 (2%)	0.75	87.25

^a^The emissions from ZnO nanoparticles were detected at 540 nm with a laser excitation wavelength of 375 nm. Numbers in parentheses indicate the relative weighting.

In case of the as-synthesized ZnO nanoparticles (hydrolyzed at 60 °C), it was observed that the fraction of electrons following the faster nonradiative decay path (τ_1_ in Table 1) increased significantly from 33% to 72% upon the addition of BR into the nanoparticle system, validating the FRET process between donor ZnO and acceptor BR. Using picosecond-resolved spectroscopy techniques we have recently demonstrated that both singly charged (V_O_^+^) and doubly charged (V_O_^++^) oxygen vacancy centers contribute to the energy transfer process with efficiencies of about 78% and 89%, respectively [15]. In the present study, the average lifetime (τ_avg_) of the fluorescence decay of the as-synthesized ZnO nanoparticles was found to be reduced by an order of magnitude upon the addition of BR, from 8.78 ns to 0.79 ns, with an energy transfer efficiency (*E*) of 91.53%.

However, upon annealing the nanoparticles, τ_avg_ was observed to be reduced in the presence of the BR molecules with a higher number of electrons following the faster decay path (τ_1_), reaching a maximum of 85% in case of the nanoparticles annealed at 250 °C, which have the maximum number of surface defects. This suggests that in nanoparticles with a higher amount of surface defect-states, more photo-excited electrons lose their energy via a nonradiative path. This loss of energy via the nonradiative path can be directly correlated to the FRET process. The population of the trap-state electrons, indicated by the number in parenthesis in the slower time component (τ_3_) of the fluorescence decay curves, was also found to be highest (25%) in the case of the ZnO nanoparticles annealed at 250 °C with the highest concentration of surface defect-states. For the various ZnO nanoparticles samples, the energy transfer efficiency (*E*) during the FRET process was then calculated using Equation 2 (see Experimental section). It was observed that the energy transfer efficiency increases with an increasing number of surface defect-states. For the ZnO nanoparticles annealed at 250 °C, which had the maximum number of surface defect-states, the highest energy transfer efficiency of 93.79% was observed, which is reduced gradually to 87.25% in the samples annealed at 350 °C, because of the reduction in the surface defects in the nanoparticles. It was observed earlier in the steady-state photoluminescence study of the annealed ZnO nanoparticles (Figure 3a), that the defect mediated green–yellow luminescence from oxygen vacancies is maximum for the nanoparticles annealed at 250 °C in air, which corroborates the highest energy transfer efficiency observed here. As a result, the ZnO nanoparticles annealed at 250 °C in air demonstrated the highest photocatalytic degradation of BR compared to the nanoparticles annealed at other temperatures. Therefore, it is clear from our results that an efficient BR degradation can be achieved through photocatalysis by using ZnO nanoparticles as the catalyst.

## Conclusion

ZnO nanoparticles with diameters of about 5–6 nm were synthesized and used as catalyst for studying the photocatalytic degradation of bilirubin. It has been demonstrated that the number of surface defect-states (mainly the oxygen vacancies) of the nanoparticles, which plays an important role in the photocatalytic degradation of the BR molecules, can be controlled through an annealing process. From picosecond-resolved TCSPC studies, it was observed that the energy related to the defect-mediated photoluminescence from the ZnO nanoparticles that peaks at about 530 nm resonantly transfers to the BR molecules adsorbed at the surface via the Förster resonance energy transfer (FRET) process: This leads to an efficient photocatalytic degradation of the BR molecules. The energy transfer efficiency of the FRET process between the annealed ZnO nanoparticles and BR molecules was estimated, and for the ZnO nanoparticles annealed at 250 °C a maximum energy transfer efficiency of more than 93% was obtained, which can be attributed to the highest concentration of surface defect-states present in these nanoparticles. Under continuous UV irradiation, the ZnO nanoparticles annealed at 250 °C showed the maximum photocatalytic activity, which was almost 3.5 times higher than that of the as-synthesized ZnO nanoparticles. In summary, from the present study, it is clear that for the efficient photocatalytic degradation of BR in blood, the defect-states in the ZnO nanoparticle catalyst, which can be modulated simply by annealing the nanoparticles, is crucial. Although DNA damage due to the exposure to UV irradiation can be a concern in case of the practical implementation of this type of FRET-based phototherapeutic systems, we believe that such harmful effects can be circumvented in the presence of the ZnO nanoparticle layer due to their ability to filter UV light. In this regard, a flow device for the removal of BR from blood, which is based on a nanoparticle film, has already been proposed in our previous study [15].

## Experimental

All chemicals used in this study were of analytical grade and used without further purification. For the synthesis of the ZnO nanoparticles, zinc acetate dihydrate (Zn(CH_3_COO)_2_·2H_2_O, Merck) and sodium hydroxide (NaOH, Merck) were used as the starting materials. Bilirubin powder was purchased from Sigma-Aldrich.

### Synthesis of ZnO nanoparticles

ZnO nanoparticles were synthesized in ethanol. In a typical synthesis process, a 2 mM Zn(CH_3_COO)_2_·2H_2_O solution was prepared in 40 mL ethanol by stirring the solution at 60 °C until a clear solution was obtained. Separately, a 4 mM NaOH solution was prepared in 20 mL ethanol by stirring the solution at 60 °C until a clear solution was obtained. Both the solutions were allowed to cool down to room temperature, then the NaOH solution was slowly added to the zinc acetate solution and mixed properly. The glass beaker was then covered tightly with aluminum foil and placed in a pre-heated water bath at 60 °C for 2 hours to hydrolyze. After 2 h, the resultant transparent ZnO nanoparticle colloidal solution was allowed to cool down to room temperature and stored in a refrigerator for further use.

#### Photocatalyst preparation

For the annealing of the ZnO nanoparticles, six glass substrates (1.5 cm × 1.5 cm) were placed on a hot plate (60 °C) and 100 μL of the as-prepared ZnO nanoparticle colloidal solution was dropped on each glass substrate. The glass substrates were initially cleaned by successive sonication in soap water, acetone, ethanol and DI water respectively for 15 min each. Once the solvent on the glass substrates dried, another 100 μL of the ZnO nanoparticle colloid was dropped again and the process was repeated several times. A total of 10 mL ZnO nanoparticle colloid was dropped on each glass substrate. Finally, the ZnO nanoparticle coated glass substrates were annealed at different temperatures up to 350 °C in air for 1 h. The annealed ZnO nanoparticles were then redispersed in chloroform by sonication for 1 h and stored in dark until further use.

#### Photocatalytic degradation of bilirubin

A 100 μM bilirubin (BR) stock solution (20 mL) was prepared in chloroform and stored at 4 °C in dark. For the photocatalytic tests, the BR solution was diluted in chloroform to obtain a concentration equal to 7 μM and used as the photocatalytic test sample. The concentration of the bilirubin solution was determined from our previous study, in which it was demonstrated that the photocatalysis of BR with ZnO nanoparticles as catalyst medium is maximum in the BR concentration range from 7 to 16.5 μM [15]. 100 μL of the ZnO nanoparticles redispersed in chloroform (0.5 μM) was then added to 2.9 mL of BR test solution in a quartz cuvette and the photocatalysis was carried out using a 12 W UV lamp. For the selective excitation of the ZnO nanoparticles, we have used a 320 nm high pass filter to block the light with wavelengths lower than 320 nm. During the photocatalysis, the degradation of BR was constantly monitored in terms of its optical absorptions recorded every two minutes by using a UV–vis spectrophotometer (Mikropack DH-2000 with USB4000 detector from Ocean Optics). BR adsorption at the surface of ZnO nanoparticles was carried out in the dark for 30 min prior to the UV light irradiation. The photocatalytic test was then carried out for 90 min and repeated thrice. BR degradation was determined by plotting the absorption peak of BR at 450 nm with respect to the UV irradiation time.

#### Langmuir–Hinshelwood kinetics model for the photocatalytic degradation of BR

The Langmuir–Hinshelwood (L–H) kinetics model was employed in order to explore the photocatalytic degradation rate at various initial concentrations of BR while keeping the concentration of the ZnO nanoparticle catalyst fixed. Mathematically the L–H model can be defined as:

[1]
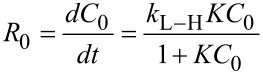


where *R_0_* is the rate of photocatalytic degradation of BR, *C*_0_ is the initial concentration of BR, *k*_L–H_ is the Langmuir–Hinshelwood (L–H) rate constant, *K* is the Langmuir adsorption coefficient of BR molecules and *t* is the UV illumination time. The values of *k*_L–H_ and *K* can be derived by considering the two boundary conditions of Equation 1. If the initial concentration of BR (*C*_0_) is sufficiently low (i.e., *KC*_0_ << 1), then Equation 1 can be rewritten as:

[3]
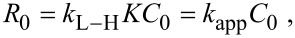


where *k*_app_ is the apparent first-order rate constant. If *C*_0_ is sufficiently high (i.e., *KC*_0_ >> 1), then Equation 1 can be simplified to a zero-order equation of the form

[4]
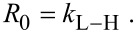


For this study the initial concentration of BR in the photocatalytic system was varied from 0 to 52.80 μM and the values of *k*_L–H_ and *K* were determined by fitting the *R*_0_ vs *C*_0_ curves using a hyperbolic function.

#### Characterization techniques

Transmission electron microscopy (TEM; model: JEOL JEM-2010) was used for the morphological and crystallographic characterization of the ZnO nanoparticles used in this study. The TEM samples were prepared by applying a drop of the ZnO nanoparticle samples dispersed in ethanol onto the carbon side of carbon-coated copper grids and dried overnight in air. The sizes of the nanoparticles were then determined by using the ImageJ software. X-ray diffraction (XRD) of the various ZnO nanoparticle samples were recorded by using a Rigaku MiniFlex 600 (Cu Kα radiation, λ =1.54 Å) XRD machine.

Steady state absorption and emission spectra of the nanoparticles were measured with an Ocean Optics Mikropack DH-2000 spectrophotometer and Jobin Yvon Fluoromax-3 fluorometer, respectively. All the photoluminescence transients were measured by using the picosecond-resolved time-correlated single photon counting (TCSPC) technique with a commercially available picosecond diode laser-pumped fluorescence spectrophotometer (LifeSpec-ps) from Edinburgh Instruments, U.K. Picosecond excitation pulses from the picoquant diode laser were used with an instrument response function (IRF) of 60 ps. A microchannel-plate photomultiplier tube (MCP-PMT, Hammamatsu) was used to detect the photoluminescence from the sample after dispersion through a monochromator. For all transients, the polarizer on the emission side was adjusted to be at 55° (the “magic angle”) with respect to the polarization axis of the excitation beam.

#### Data analysis

The picosecond resolved decay curves were fitted by using a nonlinear least square method to the function


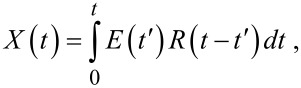


which comprises the convolution of the IRF, *E*(*t*), with a sum of exponentials


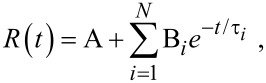


where B_i_ is the pre-exponential factor, τ*_i_* are the characteristic lifetimes and A represents a background. The relative concentration in a multi-exponential decay was expressed as:


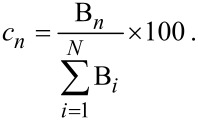


The average lifetime (amplitude-weighted) of a multi-exponential decay [32] is expressed as


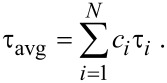


In order to estimate the energy transfer efficiency (*E*) between the ZnO donor nanoparticles and the BR acceptor molecules, the relative fluorescence lifetime of the donor in the absence (τ_D_) and presence (τ_DA_) of the acceptor were initially determined. The efficiency *E* was then calculated using the following equation:

[2]
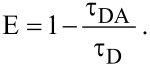


## Supporting Information

File 1 Experimental details
