# Supplementary material for: Modulation of defect-mediated energy transfer from ZnO nanoparticles for the photocatalytic degradation of bilirubin
Source: Beilstein J Nanotechnol. 2013 Nov 4;4:714–25. doi: 10.3762/bjnano.4.81 (PMC3869374; doi:10.3762/bjnano.4.81)
Supplement: File 1 — Experimental details [file Beilstein_J_Nanotechnol-04-714-s001.pdf]

Supporting Information

for

Modulation of defect-mediated energy transfer from ZnO nanoparticles for the photocatalytic degradation of bilirubin

Tanujjal Bora[1,2], Karthik K Lakshman[2], Soumik Sarkar[3], Abhinandan Makhal[3], Samim Sardar[3], Samir K Pal[3] and Joydeep Dutta*[1,2]

[1]Center of Excellence in Nanotechnology, School of Engineering and Technology, Asian Institute of Technology, P. O. Box 4, Klong Luang, Pathumthani – 12120, Thailand; [2]Chair in Nanotechnology, Water Research Center, Sultan Qaboos University, P. O. Box 17, Al Khoud – 123, Sultanate of Oman and [3]Department of Chemical, Biological & Macromolecular Sciences, Unit for Nanoscience & Technology, S. N. Bose National Centre for Basic Sciences, J D Block, Sector III, Salt Lake, Kolkata – 700 098, India

Email: Joydeep Dutta* - dutta@squ.edu.om

* Corresponding author

Experimental details

Estimation of the size of air-annealed ZnO nanoparticles

The crystallite size of the ZnO nanoparticles annealed in air at different temperatures was estimated from their respective XRD patterns by using the Scherrer equation, shown as Equation 1 below. The mean crystallite size *D* is calculated from the full-width at half-maximum (FWHM) of the XRD peak corresponding to the (102) and (104) crystal planes of the annealed ZnO nanoparticles respectively and the results are shown in Table S1.

$$D = \frac{K'\lambda}{B\cos\theta} \qquad (1)$$

where *K'* is a constant with a value about 0.9, λ is the wavelength of the X-ray (0.154 nm), *B* is the FWHM of (*hkl*) peak at diffraction angle θ.

Table S1: Mean diameter (*D*) of the ZnO nanoparticles annealed at various temperatures estimated from XRD and TEM analysis.

Annealing temperature (°C)	*D* in (102) orientation (nm)	*D* in (104) orientation (nm)	mean *D* from XRD (nm)	mean *D* from TEM (nm)
60[a]	8.3	6.2	7.3	4.5
150	8.6	6.6	7.6	4.8
200	8.2	7.2	7.7	5.1
250	8.5	7.1	7.8	5.5
300	8.2	9.1	8.7	5.7
350	8.7	8.8	8.8	5.9

[a]The 60 °C sample indicates the non-annealed as-synthesized ZnO nanoparticles.

The size of the nanoparticles was also estimated from their corresponding TEM images using ImageJ software. Figure S1 below shows the TEM images used for the estimation of the sizes of the ZnO nanoparticles annealed at different temperatures.

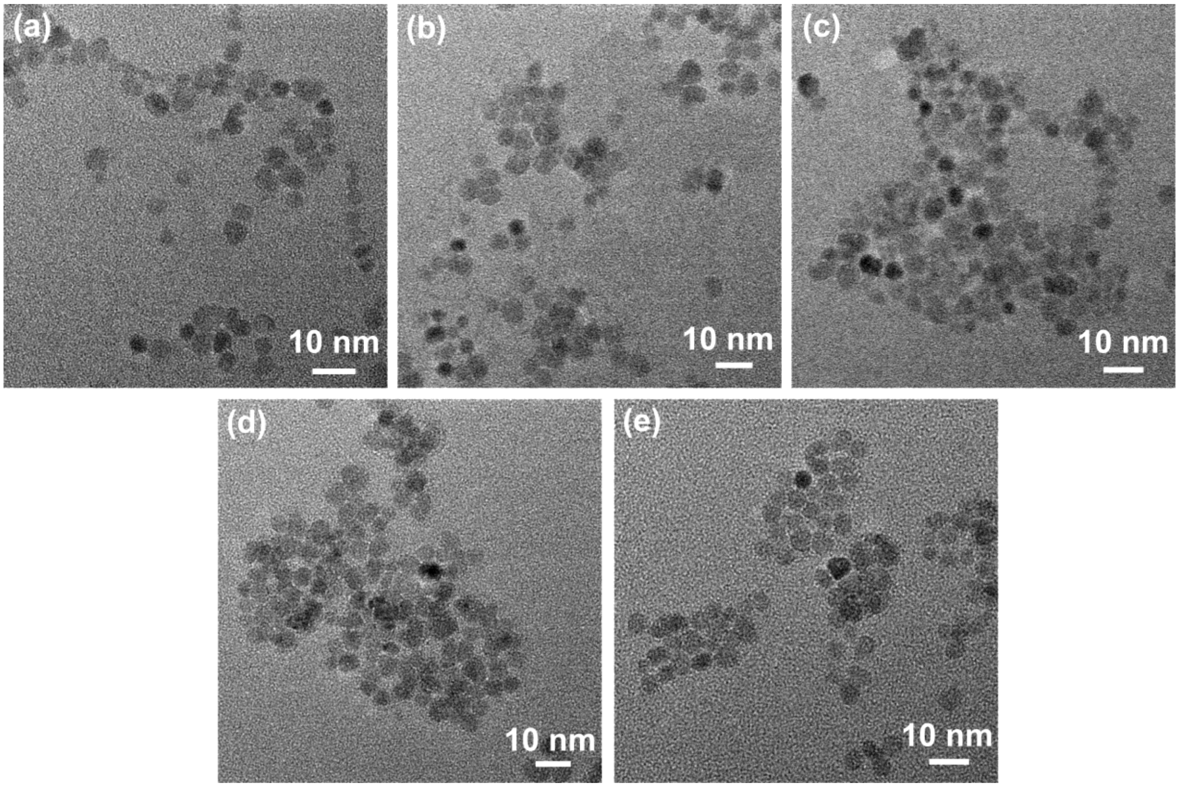

Figure S1: Transmission electron micrographs of (a) as synthesized and (b) 150 °C, (c) 200 °C, (d) 300 °C and (e) 350 °C annealed ZnO nanoparticles. The size of the ZnO nanoparticles were estimated from the respective TEM images using ImageJ software.

Adsorption of BR on ZnO

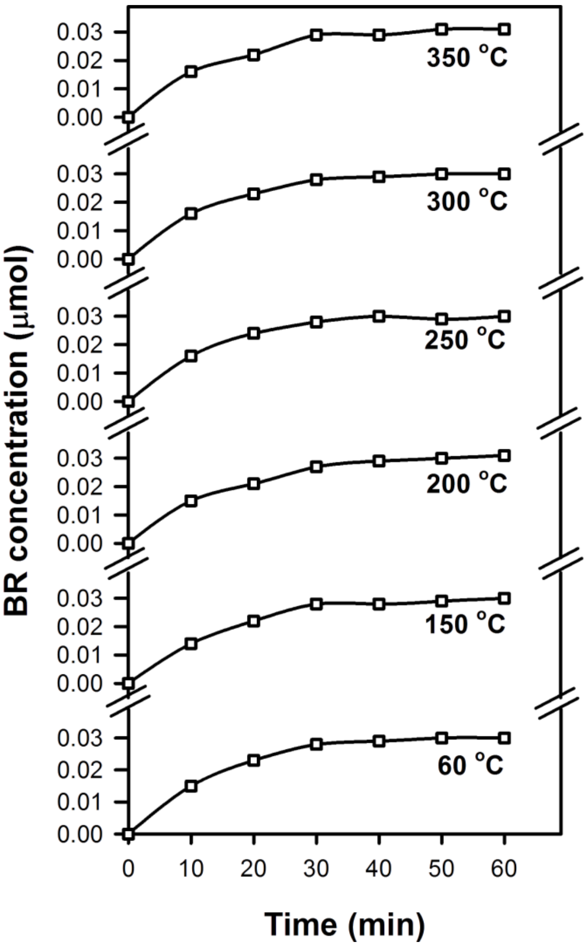

Figure S2: Kinetics of BR adsorption on ZnO nanoparticle surfaces performed in dark. The ZnO nanoparticles were air annealed at different temperatures up to 350 °C.

The kinetics of BR adsorption on the surface of the ZnO nanoparticles annealed at different temperatures performed in dark is shown in Figure S2. The concentration of the surface adsorbed BR molecules was estimated by subtracting the concentration of BR molecules present in the solution at a given time from the initial concentration of BR solution. In order to determine the concentration of BR solution C_t at a given time t, the Beer–Lambert law was used as shown below:

$$C_t = \frac{A}{\varepsilon l} \qquad (2)$$

Here, A is the BR absorbance value at 450 nm wavelength, ε is the molar extinction coefficient of BR at 450 nm (54889 $L \cdot mol^{-1} \cdot cm^{-1}$) and l is the path length, which is the width of the cuvette (1 cm).
